# Changing Fashions in Medical Treatment

**Published:** 1902-01-25

**Authors:** 


The Hospital

A JOURNAL OF

The Medical Sciences and Hospital Administration.

VOL. XXXI.—No. 800.] EDITED BY SIR HENRY BURDETT, K.C.B., AND SOLOMON C. SMITH, M.D., M.R.C.P. [JANUARY 25, 1902

### CHANGING FASHIONS IN MEDICAL TREATMENT.

It is interesting to look back thirty years and note the great changes which have taken place in the principle and practice of the art of healing during that period. The germ theory of disease was beginning to be generally understood, and pathologists, with the aid of powerful microscopes and tubes of gelatine, were investigating the habits and customs of these newly discovered and unfriendly visitors to the higher animals and man. Next the myriad armies of parasites were vigorously assaulted by various substances which were found to have the power of killing both them and their spores. This period may be described as the reign of antisepsis. Patients with bronchitis, pneumonia, phthisis and other lung troubles were made to inhale steam highly charged with vapours of benzoin or other substances, which it was hoped would arrest the action of, or destroy, the pneumococcus or tubercle bacillus in the lungs. At the same time great precautions were taken for fear an odd germ should gain access into an operation wound; the air around and often the face and clothes of the operator and his assistants being saturated with steam and carbolic acid by means of Lister's spray. This has now been superseded by what is known as the aseptic method, which aims at the prevention of access of germs, not by killing them at the time of operation but by excluding their presence from the room, the skin and the instruments. The latter are boiled, and the former rendered scrupulously clean and free from all particles of dirt or dust.

It was next discovered that it was not the mechanical action of the germ which killed its unfortunate host in most cases, but the chemical products arising from its activity which, disseminated by the blood stream, produced a poisonous effect. The physician and pathologists, therefore, sought for antidotes for these, and soon discovered that the germs, like higher forms of life, would die if forced to live on their own waste products. This led to the practice of injecting preparations resulting from the activity of germs, modified by various processes, into the patient with the hope that the living bacilli would thereby be destroyed. In the case of tubercle the results did not justify the hope, but in that of diphtheria antitoxin more favourable effects have been produced. Still another method is to inoculate the individual artificially with a modified virus and so produce a modified form of the disease and render him immune to the severe form. Broadly speaking, this was the principle adopted in the inoculation of small-pox now superseded by vaccination. Coupled with this active treatment against the germ, the body of its host was also rendered as strong as possible to resist its onslaught, and for this purpose large doses of cod liver oil and other forms of highly nutritious food stuffs were ordered in cases of wasting diseases. Light and oxygen were also found to be germicides, and hence we came to the benefit of light and airy surroundings.

Thus we reached the last stage of all which is the open-air treatment of consumption. All these researches, all this knowledge, all these complicated pathological actions have at length led to the conclusion that the best treatment is Nature's great restorer Fresh Air. The old muffling up of the body to prevent "catching cold" is to be discarded, and a patient is to exercise, sit, eat and sleep in the open air, being merely protected from rain and snow.

It is not 15 years ago since an eminent surgeon asked a member of his clinique what was the "sheet anchor" of surgery, and the student, after thinking for a minute, answered, "Iodide of potassium." "No," replied the teacher; "the sheet anchor of surgery is Rest." Fractures, sprains and most other forms of injury were then treated by rendering the part immobile for weeks with plaster of Paris or other forms of splints. A great change has since taken place. Passive movements and massage are now commenced, in all cases involving an injury to a joint, a few days after the accident. The result is that cases of impaired mobility are becoming a thing of the past and the necessity of breaking down adhesions is unknown in good modern practice. In medicine also, exercise is not merely ordered as a common sense treatment, but old gentlemen and ladies are taught definite movements to cause their sluggish livers to act, and all kinds of appliances, such as artificial horses, are to be procured for this purpose.

Young women who would otherwise suffer from constipation, anæmia, and many other complaints now perform a variety of exercises, with or without the aid of machinery, before their morning bath, and spend a certain portion of the day in gymnastics and fencing. We hope, therefore, that the rising generation of children will be the offsprings of robust and clean—physically and mentally—mothers, and will be brought up in a similar manner. The advice, "Fresh air and exercise" is as old as the hills, but now it is "rational," not empirical, now its action is thought to be understood, not merely supposed.

Nor can we argue that all our knowledge is superfluous, notwithstanding the constant changes in its teachings, and that no advance has been made because many steps have seemed to lead to error. The greater the knowledge the simpler appears the most complicated of Nature's phenomena, and the time may come when the whole of therapeutics may be literally summed up in "Fresh air and exercise."

